# Comparison of European Standard Patch Test Results of 330 Patients from an Occupational Diseases Hospital

**DOI:** 10.1155/2016/9421878

**Published:** 2016-10-11

**Authors:** Özge Gündüz, Aslı Aytekin, Engin Tutkun, Hınç Yılmaz

**Affiliations:** ^1^Ufuk University Faculty of Medicine, Department of Dermatology, Ankara, Turkey; ^2^Department of Dermatology, Ankara Occupational Diseases Hospital, Ankara, Turkey; ^3^Department of Occupational Diseases, Ankara Occupational Diseases Hospital, Ankara, Turkey

## Abstract

*Background and Aim.* Contact dermatitis (CD) is the most prevalent occupational skin disease with a significant impact on quality of life. Patch testing is used for the identification of responsible allergens which may improve protective and preventive measures in the workplace. Herein, we aim to identify the demographic characteristics and occupation of patients with early diagnosis of occupational CD and compare patch test results.* Materials and Methods.* The study included 330 patients referred to our clinic between April 2009 and April 2011 and who were patch-tested with 28-allergen European Standard Test.* Results.* 126 (38%) patients were female and 204 (62%) were male with a mean age of 36.12 (±13.13) years. Positive allergic reactions were observed in 182 (55%) patients. Nickel sulphate (41/126) and potassium dichromate (39/204) were significantly the most common allergens in women and men, respectively (*P* < 0.005). Additionally, the most common occupation in women was household activities (83/126) and in men was manufacturing (80/204).* Conclusion.* The allergens to which people become sensitized differ according to their working environment and occupation. Classification of occupations is important for identification of sensitization risks and monitoring of changes in allergen distribution of different occupations.

## 1. Introduction

Contact dermatitis (CD) is the most prevalent occupational skin disease, comprising 90% of reported job-related cases [1]. Occupational CD (OCD) may necessitate sick leave and has been shown to have a significant impact on quality of life [2]. Eighty percent of all OCD cases are attributed to irritant CD and the remaining to allergic CD [3, 4]. Contrary to irritant CD, allergic CD is mediated by a delayed-type hypersensitivity reaction which can be shown on patch testing. Patch testing is an important diagnostic tool for the identification of allergens responsible for dermatitis and the differentiation between allergic and irritant CD. However, in reality, the causes of allergic OCD are often multifactorial in origin and various irritating factors in the working environment may contribute to the penetration of allergens into the skin.

In our country, The Occupational Diseases Hospital specializes in making medicolegal decisions regarding occupational diseases and can be attended by all workers throughout the country. Patients are evaluated in the hospital's Occupational Diseases Policlinic by physicians with expertise in occupational diseases. Additionally, local patients may also visit the Dermatology Outpatient Clinic.

In this study, we aim to identify the demographic characteristics and occupation of patients who had attended the Dermatology Policlinic of an Occupational Diseases Hospital with early diagnosis of OCD and compare the patch test results with these variables.

## 2. Materials and Methods

This retrospective descriptive study included 330 patients who visited or were referred to the Dermatology Policlinic between April 2009 and April 2011. After referral to the Dermatology Clinic, full clinical and thorough occupational history was taken and physical examination and subsequent investigation using patch testing were made for the diagnosis of occupational contact dermatitis. Patch tests were performed on all patients according to the European Standard Series (ESS) of 28 allergens using IQ Chambers (Chemotechnique Diagnostics, Sweden). Patients were not tested with additional allergens. In line with the clinic's protocol, patch tests were applied to patients' back for a duration of 48 hours. Readings were made at the 48th, 72nd, and 96th hours in accordance with International Contact Dermatitis Research Group (ICDRG) guidelines. An erythematous reaction limited to the application site, which usually fades within 96 hours, and follicular pustules were considered as irritant reactions. Patients were tested one week after discontinuation of antihistamines and topical corticosteroid therapy and at least four weeks after cessation of immunosuppressive agents. Pregnant and lactating women were not included.

The patients were questioned about their job, working environment, protective measures, and hobbies or spare-time activities. The final diagnosis was made after clinical examination, patch testing, and assessment of clinical relevance which were evaluated based on questioning the patient regarding the relation between the work process and exposure of skin to allergens; medical files and material safety data sheets were collected from occupational physician. Patient files were retrospectively reviewed and demographic properties recorded. This study was approved by local ethics committee (22/02/2012-20).

Predictive Analytics Software (PASW) version 18.0 for Windows (SPSS Inc., Chicago, IL, USA) was used for all analyses. The single-sample Kolmogorov-Smirnov test was performed to determine data distribution and continuous variables were compared with Students'* t*-test and categorical variables were compared with the Chi-square test. A significance level of 0.05 was used for all analysis.

## 3. Results

Of the 330 patients, 126 (38%) were female and 204 (62%) were male. Mean age was 36.12 (±13.13) years. There was a history of atopy in 99 (30%) patients. Patients' most common complaint was pruritus (23%). The most frequently affected areas were the hands alone (53%), the face and neck (14%), and the hands and feet combined (8%).

One (22%) or more than one (33%) positive allergic reactions were observed in 182 (55%) patients. The most common allergens causing positive reactions were nickel sulphate (*n* = 63, 19%), potassium dichromate (*n* = 48, 14.5%), and cobalt chloride (*n* = 43, 13%). The least common allergens were wool alcohols (*n* = 1), N-isopropyl-N-phenyl-4-phenylenediamine (*n* = 2), and Quaternium (*n* = 2). Among all the tested patients, positive reactions were noted to all allergens in the ESS. Nickel sulphate and potassium dichromate were significantly the most common allergens in women and men, respectively (Table 1). There was no significant difference in multiple allergen positivity between women (37%, *n* = 47) and men (27.9%, *n* = 57) (Chi-square test, *P* = 0.13).

**Figure 1:**
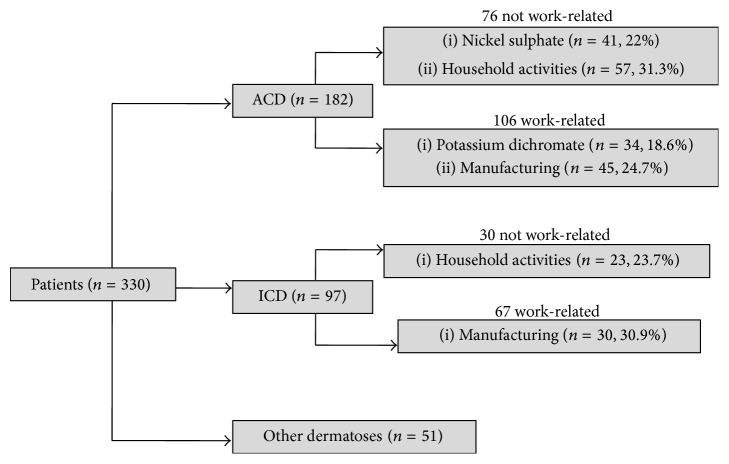
Final diagnosis after determination of clinical relevance.

The final diagnosis after clinical examination, patch testing, and assessment of clinical relevance are presented in Figure 1. The distribution of patients according to Statistical Classification of Economic Activities in the European Community (NACE) codes is shown in Table 2.

## 4. Discussion

CD mainly affects the exposed areas of the body such as the hands, face, and neck. Similarly, these areas were the most frequently affected sites in our study. Although the list of top allergens varies between countries, nickel sulphate is the most commonly reported sensitizer in most parts of the world (Table 3). Sensitization rates differ from one country to another, ranging from 13.8% to 24.4% [5–12]. In previous studies conducted in Turkey, the frequency of positive patch test reactions were reported as 32.3% and 51.6% [13, 14]. Although in the present study our population is relatively small, one or more positive allergic reactions were observed in 55% of patients, which might be due to the fact that this study was conducted in an occupational diseases specified hospital where the study population was composed of patients who might be exposed to multiple different allergens in the work environment. Similar to the studies of Akasya-Hillenbrand and Özkaya*-*Bayazit and Akyol et al., which reported nickel sensitivity of 17.6% and 19.1%, respectively, the most common allergens causing positive reactions in our study were nickel sulphate (19%), potassium dichromate (14.5%), and cobalt chloride (13%) [13, 14]. Although, potassium dichromate and cobalt chloride sensitivity was similar in our study (14.5% and 13%, resp.) to that of Akasya-Hillenbrand and Özkaya*-*Bayazit (11.8% and 8.5%, resp.), our results were more frequent than those of Akyol et al. (4.6% and 5.3%, resp.) [13, 14]. The reason for this difference might be due to location: Akasya-Hillenbrand and Özkaya*-*Bayazit's study was conducted in the highly industrialized city of Istanbul whereas Akyol et al.'s study was held in Ankara, a governmental city [13, 14]. This also favors the fact that the variation in the occupations of study populations leads to various allergen exposures in the work environment and the difference in patch test results. In addition, the fact that nickel and/or chromate sensitivity was accompanied by cobalt sensitivity was expected.

In terms of allergic reaction frequency, no difference was found between men (51%) and women (62%) (*P* = 0.085). However, the most common allergens in which women and men became sensitized to are significantly different (Table 1). Similarly, nickel allergy is reported to be the most common allergen among young women, and ear piercing was a common risk factor for developing nickel allergy while CD caused by chromate sensitivity is reported to be more common in men and is thought to be caused by occupational exposure to soluble compounds in cement or leather [15, 16]. Potassium dichromate is found in cement, textile inks, paints, varnishes, and leather processes, whereas thiuram mix, mercapto mix, and mercaptobenzothiazole are rubber accelerators which are components of both natural and synthetic rubber and are also found in gloves [17]. Additionally, female patients were significantly more frequently sensitized by fragrance mix (*n* = 19) and balsam of Peru (*n* = 13); both are found in perfumes and cross react with each other. The difference in sensitization between men and women might be due to the fact that most common occupations in women and men were household activities (83/126) and manufacturing (80/204), respectively. Therefore, men might be exposed to multiple and various allergens with inadequate protective measures in their working environment. Additionally, significant rubber accelerator allergy in working men might be also due to the use of protective gear (i.e., gloves and rubber boots) during working hours. Unfortunately, sensitivity to chromium and nickel is related to a worse prognosis of occupational ACD as these allergens are ubiquitous in the environment and are therefore difficult to avoid [3].

In our study of 330 patients, 52% were diagnosed as having OCD. Allergic OCD affected 106 (61.3%) patients, and irritant OCD was found in 67 (38.7%). Although our study was comprised of a limited number of patients, it is the first study focused on OCD from an Occupational Diseases Hospital; therefore it represents a wide range of patients with different occupations throughout the country.

NACE is used for the classification of economic activities in the European Union since 1970. Classification of occupations according to NACE enable us to enlighten the distribution of risk groups. In NACE classification, housewives and retirees (*n* = 100) comprised the most common occupational group, followed by manufacturing (*n* = 85). Those working in the manufacturing area also constituted the majority of those diagnosed with occupational ACD or ICD (Figure 1). Potassium dichromate was the most common allergen in those working in the sectors of manufacturing (10/45) and construction (11/17) whereas nickel sulphate was most common in patients in the household (33/59) and education (9/17) groups. The major sources of chromate exposure are construction materials (cement, drywall), leather, and metal-working occupations (welding, plating, and dyeing) [18]. Also the working environment can be easily contaminated by cement dust or chromate containing solutions, which makes the prognosis for the chromate-sensitized patient poor [19]. Therefore, those patients who were diagnosed as occupational ACD were suggested a change of job. Household and education groups were mainly composed of women who were most frequently sensitized by nickel. Additionally, wet work, such as in household activities, causes skin barrier damage and increases the risk of developing hand eczema for individuals with nickel allergy [20].

The main limitation of our study was the relatively small population. Additionally, two different dermatologists performed readings and interpretations of patch tests and did not check each other's work. The fact that patients were evaluated by different clinicians may be considered as another limitation of this study.

In conclusion, this study shows that allergens to which people are sensitive differ according to their working environment and type of occupation. We would like to emphasize that classification of occupations using an international standard such as the NACE Coding is important for the identification of sensitization risks of different occupations, the comparison of study results, and the monitoring of changes in allergen distribution of different occupations.

## Figures and Tables

**Table 1:** Comparison of patch test results of men and women.

	Women (*n* = 126)	Men (*n* = 204)	*P*
Age (years)	33,31	37,12	0.372
Positive reaction	78	104	0.085

*Allergens*			
Potassium dichromate	9	39	0.005
Rubber accelerator	1	19	0.001
Cobalt(II) chloride	24	19	0.007
Nickel sulphate	41	22	<0.001
Fragrance mix I and II	19	12	0.037
Balsam of Peru	13	8	0.034

*Occupations*			
	Household activities *n* = 83 (65.8%)	Manufacturing *n* = 80 (39.2%)	

Rubber accelerator: thiuram mix, mercapto mix, and mercaptobenzothiazole.

**Table 2:** Comparison of occupations according to NACE codes and patch test positivity.

Occupations	# of patients	# of positive results	Most common allergens (*n*)
Agriculture, forestry, and fishing	1	1	
Mining, quarrying	4	3	
Manufacturing	85	45	Potassium dichromate (10)
Electricity, gas, steam and air conditioning supply			
Water supply, sewerage, waste management, and remediation activities			
Construction	29	17	Potassium dichromate (11)
Wholesale and retail trade, repair of motor vehicles and motorcycles	27	11	Cobalt(II) chloride (5)
Transportation and storage	5	3	
Accommodation and food service activities	3	1	
Information and communication			
Financial and insurance activities			
Real estate activities			
Professional, scientific, and technical activities	9	6	
Public administration and defence, compulsory social security	4	2	
Education	34	17	Nickel sulphate (9)
Human health and social work activities	9	6	
Arts, entertainment, and recreation	1	0	
Other service activities	19	11	Potassium dichromate (4)
Activities of households as employers, undifferentiated goods- and services- producing activities of households for own use	100	59	Nickel sulphate (33)
Activities of extraterritorial organisations and bodies			
*Total*	330	182	

NACE: Nomenclature des Activités Économiques dans la Communauté Européenne (Statistical Classification of Economic Activities in the European Community); #: number.

**Table 3:** Most common allergens in previous studies.

	Country	# of pt	# of positive reactions (%)	Most common 3 allergens (%)		
Our results	Turkey	330	182 (55%)	Nickel sulphate (19%)	Potassium dichromate (14.5%)	Cobalt chloride (13%)
Akasya-Hillenbrand and Özkaya-Bayazit [13]	Turkey	542	280 (51.6%)	Nickel sulphate (19.1%)	Potassium dichromate (11.8%)	Palladium chloride (9.4%)
Akyol et al. [14]	Turkey	1038	336 (32.3%)	Nickel sulphate (17.6%)	Cobalt chloride (5.3%)	Potassium dichromate (4.6%)
Beliauskiene et al. [5]	Lithuania	816	384 (47.4%)	Nickel sulphate (16.4%)	Balsam of Peru (8.6%)	*p*-Phenylenediamine (5.8%)
Bilcha et al. [6]	Ethiopia	514	271 (52.7%)	Nickel sulphate (17.7%)	Fragrance mix I (14.8%)	Cobalt chloride (8.0%)
Lam et al. [7]	Hong Kong	2585	1415 (54.7%)	Nickel sulphate (24.4%)	Fragrance mix (13.7%)	Cobalt chloride (8.7%)
Lazarov [8]	Israel	2156	937 (43.5%)	Nickel sulphate (13.9%)	Fragrance mix (7.1%)	Potassium dichromate (3.8%)
Wetter et al. [9]	USA	1324	917 (69.3%)	Nickel sulphate (14.1%)	Balsam of Peru (11.3%)	Neomycin sulphate (11.2%)
Machovcova et al. [10]	Czech Republic	12058	7661 (63.5%)	Nickel sulphate (13.7%)	Balsam of Peru (7.28%)	Fragrance mix (5.7%)
Lestringant et al. [11]	United Arab Emirates	373	224 (60%)	Nickel sulphate (15%)	Fragrance mix (8.0%)	PTBP (7.5%)

#: number; pt: patients; USA: United States of America; PTBP: p-tert-butylphenol formaldehyde resin.
